# Involvement of the bone morphogenic protein/SMAD signaling pathway in the etiology of congenital anomalies of the kidney and urinary tract accompanied by cryptorchidism

**DOI:** 10.1186/s12894-017-0300-9

**Published:** 2017-12-02

**Authors:** Kentaro Mizuno, Akihiro Nakane, Hidenori Nishio, Yoshinobu Moritoki, Hideyuki Kamisawa, Satoshi Kurokawa, Taiki Kato, Ryosuke Ando, Tetsuji Maruyama, Takahiro Yasui, Yutaro Hayashi

**Affiliations:** 1 Department of Nephro-urology, Nagoya City University Graduate School of Medical Sciences, Nagoya, Japan; 2 Department of Pediatric urology, Nagoya City University Graduate School of Medical Sciences, 1 Kawasumi, Mizuho-cho, Mizuho-ku, Nagoya, Japan

**Keywords:** Congenital anomalies of the kidney and urinary tract, Cryptorchidism, Exome sequencing, Renal development

## Abstract

**Background:**

Congenital anomalies of the kidney and urinary tract (CAKUT), such as renal dysplasia, hydronephrosis, or vesicoureteral reflux, are the most common causes of end-stage renal disease. However, the genetic etiology of CAKUT remains unclear. In this study, we performed whole exome sequencing (WES) to elucidate the genetic etiology of symptomatic CAKUT and CAKUT accompanied by cryptorchidism.

**Methods:**

Three patients with unilateral renal dysplasia accompanied by ipsilateral cryptorchidism were included in this analysis. Genomic DNA was extracted from peripheral blood, and WES was performed. Disease-specific single nucleotide polymorphisms (SNPs) were determined by comparison with the human genome reference sequence (hg19). Additionally, we searched for SNPs that were common to all three patients, with a particular focus on the coding regions of the target genes.

**Results:**

In total, 8710 SNPs were detected. Of the genes harboring these SNPs, 32 associated with renal or testicular development were selected for further analyses. Of these, eight genes (i.e., *SMAD4*, *ITGA8*, *GRIP1*, *FREM1*, *FREM2*, *TNXB*, *BMP8B*, and *SALL1*) carried a single amino acid substitution that was common to all three patients. In particular, SNPs in *SMAD4* (His290Pro and His291Pro) have not been reported previously in patients with symptomatic CAKUT. Of the candidate genes, four genes (i.e., *ITGA8*, *GRIP1*, *FREM1*, and *FREM2*) were Fraser syndrome-related genes, encoding proteins that functionally converged on the glial cell-derived neurotrophic factor/RET/bone morphogenic protein (BMP) signaling pathways. As another candidate gene, the protein encoded by *BMP8B* activates the nuclear translocation of SMAD4, which regulates the expression of genes associated with the differentiation of primordial germ cells or testicular development. Additionally, BMP4, a member of the BMP family, regulates the interaction between metanephric mesenchyme and ureteric buds by suppressing GDNF.

**Conclusions:**

Taken together, our findings suggested that the development of the kidney and urinary tract is intimately linked with that of male reproductive organs via BMP/SMAD signaling pathways.

## Background

Congenital anomalies of the kidney and urinary tract (CAKUT) account for approximately 40–50% cases of end-stage renal disease in children [1]. CAKUT is a comprehensive disease concept, including renal hypoplasia, dysplasia, hydronephrosis (ureteropelvic junction obstruction or ureterovesical junction obstruction), ureter duplex, vesicoureteral reflux, and posterior urethral valves [2]. The incidence of CAKUT is estimated to be 3–6 per 1000 live births [3]. In general, the structural anomalies found in patients with CAKUT are often related because impaired codevelopment of nephrogenic tissues derived from the metanephric mesenchyme and ureteric bud is thought to contribute to the pathogenesis of CAKUT [4]. Thus, the etiology of CAKUT is heterogeneous and includes mutations in genes involved in the embryonic development of the kidneys [2]. A previous study showed that monogenic causes could be detected in about 12% of patients [5]. Thus, elucidating the genetic etiology of CAKUT is important for the diagnosis of asymptomatic renal disease, advanced diagnosis of inheritance patterns, and referral to genetic counseling in the clinical setting [2].

While CAKUT most frequently occurs as an isolated case and without renal or urinary tract structural anomalies or symptoms, it may also appear with other systemic disorders, such as Fraser syndrome [1], Hirchsprung disease [6], or vertebral defects, anal atresia, cardiac defects, tracheo-esophageal fistula, renal anomalies, and limb abnormalities (VACTERL association) [7]. Notably, anomalies in the epididymis are sometimes encountered during surgery for cryptorchidism. Although the complete mechanism of testicular descent remains unclear, a recent study revealed that maldevelopment of the epididymis is associated with problems in testicular descent [8]. Because both the epididymis and ureteric bud are embryologically derived from the mesonephric duct, we hypothesized that a common mechanism may be responsible for the maldevelopment of the ureteric bud and impaired testicular descent. A previous study demonstrated that the pathogenesis of CAKUT is associated with mutations in several genes, including *HNF1B*, *PAX2*, or *SALL1* [9]. In contrast, studies in knockout mice [10, 11] and genome-wide association studies [12] have shown that *INSL3* and *TGFBR3* are involved in the onset of cryptorchidism. However, few reports have described the genetic etiology of CAKUT accompanied by cryptorchidism [13].

Thus, in the present study, whole-exome sequencing (WES) was used to elucidate the genetic etiology of “symptomatic CAKUT,” i.e., CAKUT accompanied by cryptorchidism. We identified single nucleotide polymorphisms (SNPs) common to all patients and detected 10 non-synonymous SNPs from eight genes known to be associated with CAKUT or testicular development, including *BMP8B* and *SMAD4*, whose gene products function in the same signaling pathway. Our findings provide important insights into the relationship between renal and testicular development and these signaling pathways.

## Methods

### Patients and sample preparation

From patients who were treated or followed-up at Nagoya City University Hospital from July 2011 to January 2015, three patients with CAKUT complicated by ipsilateral cryptorchidism were enrolled in this study. Patient characteristics are shown in Table 1. At the initial visit, every patient was referred to our hospital because scrotal contents were absent and abdominal ultrasonography, computed tomography (CT) scanning, or magnetic resonance imaging (MRI) examinations revealed renal aplasia or multicystic dysplastic kidney. Case 3 had a pelvic dysplastic kidney and underwent ipsilateral nephrectomy simultaneously with orchiectomy for abdominal testis. Chromosome analysis was performed for all patients; case 3 showed chromosomal abnormality (46,Y, add (X) (p22.3)). Genomic DNA was extracted from the peripheral blood of the patients using a Wizard genomic DNA purification kit, according to the manufacturer’s instructions, as previously reported [14]. The purity of the extracted DNA was determined by measuring the absorbance and visually by gel electrophoresis.

**Table 1:** Patient characteristics

Case No.	Diagnosis	Age	G-banding	Treatment
1	Right renal aplasia, Right abdominal testis, Agenesis of right vas deferens and seminal vesicle	31 y	46,XY	Laparoscopic right orchiopexy
2	Left multicystic dysplastic kidney, Bil. cryptorchidism, Micropenis	1 y	46,XY	Laparoscopic bilateral orchiopexy
3	Left multicystic dysplastic kidney (pelvis), Left abdominal testis, Agenesis of left vas deferens	2 y	46,Y, add(X) (p22.3)	Laparoscopic left nephrectomy left orchiectomy

### WES analysis

Whole exons were purified from genomic DNA using a SureSelevtXT Human All Exon v5 Kit (Agilent Technologies, Santa Clara, CA, USA). After ligation of specific adaptors for exon fragments, paired-end sequencing was performed on a Hiseq 2500 instrument (Illumina Inc., San Diego, CA, USA). Raw sequence data in fastq format were uploaded to the DNAnexus platform server (DNAnexus Inc., San Francisco, CA, USA) and aligned to the human reference genome (hg19). After all data were exported to Microsoft Excel and Access, we searched for SNPs that were common to all three patients, with a particular focus on the coding region.

### SNP analysis at specific gene loci

Next, we selected 32 genes that have been reported to be associated with CAKUT or testicular development: *BMP7*, *CDC5L*, *CHD1L*, *GATA3*, *HNF1B*, *PAX2*, *RET*, *ROBO2*, *SALL1*, *SIX2*, *SIX5*, *EYA2* [9], *FRAS1*, *FREM2*, *GRIP1*, *FREM1*, *ITGA8*, *GREM1*, *ILK*, *LIN7C*, *DACT1* [1], *TNXB* [15], *DSTYK* [16], *WNT4*, *WT1*, *PAX7* [17], *SALL4* [18, 19], *BMP4*, *SMAD4* [20], *BMP8B* [21], *GDNF*, and *GFRA1* [13]. From the WES data, we evaluated SNPs in these 32 genes. We further examined the shared SNPs among 3 cases using by subsequent filtering of variants based on their frequencies (minor allele frequency (MAF)) in databases including Exome Aggregation Consortium (ExAC), 1000 Genome Project, Exome Variant Server (ESP).

### Ethics statement

Studies using human genomic material were performed only after obtaining written informed consent from the patient (case 1) and the families of the patients and approval from the Nagoya City University Hospital review board (approval no. 184).

## Results

**Fig. 1:**
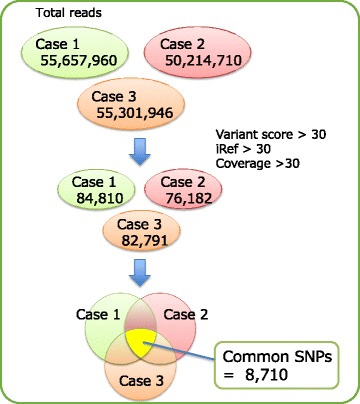
Filtering scheme for identification of common SNPs in three patients with CAKUT accompanied by cryptorchidism. The number of SNPs in each patient was decreased at each step

**Fig. 2:**
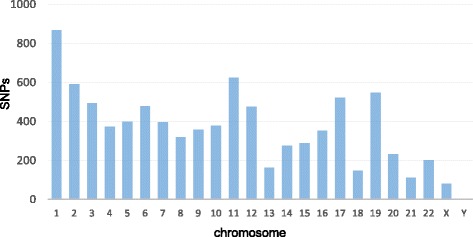
Graphical view of the number of SNPs in each chromosome. The number of SNPs was greater in chromosomes 1, 11, 12, 17, and 19 than in the other chromosomes. Chromosome Y had only two SNPs

The total number of sequencing reads for all exons of the three patients (cases 1, 2, and 3) were 55,657,960, 50,214,710, and 55,301,946, respectively. To improve the accuracy of the SNP data, raw data were filtered using the DNAnexus platform server under the following conditions: variant score > 30, iRef >30, and coverage >30. Subsequently, the numbers of SNPs in cases 1, 2, and 3 were decreased to 84,810, 76,182, and 82,791, respectively. In total, 8710 SNPs were common to all three patients (Fig. 1). The chromosomal locations of these SNPs were determined; chromosomes 1, 11, 12, 17, and 19 showed the highest number of SNPs. Chromosome Y harbored only two SNPs (Fig. 2).

We further investigated the SNPs within 32 specific gene loci and detected 10 non-synonymous SNPs within eight genes (Table 2). Of these SNPs, two in *SMAD4* had not been reported previously, suggesting that these may be novel variants in patients with CAKUT. *SMAD4* is a central mediator of the transforming growth factor (TGF)-β/bone morphogenic protein (BMP) signaling pathway and promotes transcriptional activation of target genes [22]. We also detected an SNP in *BMP8B* (rs179472; Ser276Thr). *BMP8B* belongs to the TGF-β superfamily and triggers the phosphorylation of intracellular receptor-regulated SMADs (R-SMADs), which function as transcription factors. Phosphorylated R-SMAD interacts with SMAD4 and translocates to the nucleus, where it regulates the transcription of over 500 target genes [23]. SNPs in *ITGA8*, *GRIP1*, *FREM1*, and *FREM2* have been reported in a WES study of patients with isolated CAKUT [1]; however, these findings were not consistent with our results. In addition, although a deleterious heterozygous mutation in *TNXB* (Thr3257Ile) has been shown to cause hereditary vesicoureteral reflux (VUR) [15], this was not true for the SNP detected in the present study (rs6457477; Arg504His). Moreover, the SNP identified in *SALL1* (rs4614723; Val1178Ile) was different from that reported in a WES study for familial CAKUT [9]; however, the same SNP was reported in a previous case report [24]. MAF of SNPs in *SMAD4* and *FREM2* (rs2496423) is lower than 1%, and remaining 7 SNPs in 7 genes are more common in the general population.

**Table 2:** Genes and SNPs common to patients with CAKUT accompanied by cryptorchidism

Gene Symbol	Gene Name	Gene ID	Chromosomal location	RefSeq	SNPs	SNP ID	Amino acid replacement
SMAD4	SMAD family member 4	4089	chr18: 48584791	A	C	–	His290Pro
			chr18: 48584794	A	C	–	His291Pro
ITGA8	Integrin a8	8516	chr10: 15573050	A	G	rs1041135	Val979Ala
GRIP1	Glutamate receptor interacting protein 1	23426	chr12: 66786091	G	C	rs7970387	Gln822Glu
FREM2	FRAS1 related extracellular matrix protein 2	341640	chr13: 39263714	T	C	rs2496423	Ser745Pro
			chr13: 39,430,314	C	T	rs9548509	Thr2326Ile
FREM1	FRAS1 related extracellular matrix protein 1	158326	chr9: 14737506	T	G	rs10961689	Gln2143Pro
TNXB	Tenascin XB	7148	chr6: 31977391	C	T	rs6457477	Arg504His
BMP8B	Bone morphogenetic protein 8b	656	chr1: 40230336	C	G	rs179472	Ser276Thr
SALL1	Spalt-like transcription factor 1	6299	chr16: 51171175	C	T	rs4614723	Val1178Ile

## Discussion

In the present study, we performed WES of samples from three patients with CAKUT accompanied by cryptorchidism to elucidate the genetic etiology of the disease. We detected 10 non-synonymous SNPs in the coding regions of eight genes. In particular, we identified two novel SNPs in *SMAD4* (His290Pro and His291Pro) and one SNP in *BMP8B* (rs179472; Ser276Thr). Both *BMP8B* and *SMAD4* function in the same signaling pathway, and it is likely that this pathway is involved in the etiology in CAKUT accompanied by cryptorchidism. The other candidate SNPs (*ITGA8*, *GRIP1*, *FREM1*, *FREM2*, *TNXB*, and *SALL1*) identified in this study were consistent with previous WES studies examining isolated or familial CAKUT [1, 9, 15, 24]. In particular, four of these genes (i.e., *ITGA8*, *GRIP1*, *FREM1*, and *FREM2*) were Fraser syndrome-related genes reported previously by Kohl et al. [1]. Fraser syndrome is a rare autosomal-recessive disorder with features of cryptophthalmos, syndactyly, ambiguous genitalia, laryngeal and genitourinary malformations, oral clefting, and mental retardation [25]. Because such mutated Fraser syndrome-related genes encode proteins that functionally converge on the glial cell-derived neutotrophic factor (GDNF)-RET/BMP signaling pathways at the interface of the ureteric bud and metanephric mesenchyme [1], these results also supported our hypothesis that alterations in the BMP/SMAD signaling pathway may cause renal maldevelopment with ipsilateral cryptorchidism. In case 3 patient, he has chromosomal abnormality (46,Y, add(X)(p22.3)). This chromosomal abnormality means addition of unknown fragment to terminal end of the short arm of X chromosome. Although there is the possibility that this chromosomal abnormality provide cause of his phenotype, there are no common SNPs in X chromosome among 3 cases.

In humans, the metanephros is the structure that develops into the kidneys. This structure begins to form as the ureteric bud at about 4 weeks of gestation [26]. The ureteric bud invades the metanephric mesenchyme and undergoes recursive branching to form the collecting system of the urinary tract [13]. The key regulators of primary ureteric bud outgrowth and branching are GDNF, which is secreted by the metanephric mesenchyme, and its receptor RET, which is expressed on the ureteric bud [27]. Following the binding of RET, a receptor tyrosine kinase, to the GDNF coreceptor GDNF family receptor α-1 (GFRA1), GDNF activates the RET/GFRA1 receptor tyrosine kinase and thereby triggers a signaling cascade that includes the extracellular signal-regulated kinase (ERK), phosphoinositol 3-kinase (PI3K), and phospholipase (PLC) ζ pathways [13]. Activation of these pathways eventually leads to the outgrowth of the ureteric bud and subsequent renal development [27]. As an upstream regulator of the GDNF-RET/GFRA1 signaling pathway, BMP4 acts as a negative regulator [28]. Moreover, as members of the TGF-β superfamily, BMPs regulate gene expression by receptor-mediated activation of SMAD transcription factors [23]. Therefore, the BMP/SMAD and GDNF-RET/GFRA1 signaling pathways are intimately involved in the development of the ureteric bud, which is derived from the metanephric duct, during the early phase of renal development.

Several studies have reported the role of the BMP/SMAD signaling pathway in testicular development. *BMP4* and *BMP8B* are important for the specification of primordial germ cells during fetal development [29], and *SMAD4* and *SMAD3* are necessary for the proliferation and maturation of fetal Sertoli cells [29]. The GDNF-RET/GFRA1 signaling pathway is also associated with spermatogenesis, and GDNF is essential for the self-renewal and differentiation of spermatogonial stem cells [30]. Cryptorchidism is a multifactorial disease, and its etiology is linked to multiple genomic loci as well as maternal and/or environmental factors. The genomic loci and pathways involved in its etiology remain unclear; however, Barthold et al. recently reported a phenotype-specific association of the *TGFBR3* locus with nonsyndromic cryptorchidism [12]. Furthermore, *SMAD4* mutations have been identified in patients with Myhre syndrome, which features cryptorchidism [31].

In the present study, non-synonymous SNPs in both *BMP8B* and *SMAD4* loci were found to be common to all patients with CAKUT accompanied by cryptorchidism, suggesting that impairment of the BMP/SMAD signaling pathway was likely involved in the pathogenesis of this condition. Furthermore, because renal anomalies and cryptorchidism were ipsilateral in all our patients, it is likely that development of the ipsilateral metanephric duct was impaired in these patients. CAKUT is a comprehensive disease concept that encompasses a wide array of phenotypes. To elucidate the genetic etiology of CAKUT, it will be necessary to analyze a greater number of cases. Indeed, several studies on isolated or familial CAKUT have been planned and reported [1, 2, 5, 9, 13]; however, narrowing down the patient setting can reduce the number of cases that need to be studied in order to determine the associated gene loci.

To date, several WES studies have been reported, with the aim of elucidating the genetic cause of CAKUT [5, 32–34]. Consequently, mutations in the *ZBTB24*, *WFS1*, *HPSE2*, *ATRX*, *ASPH*, *AGXT*, *AQP2*, *CTNS*, *PKHD1* [5], *TBX18* [32], *SLIT2*, *SRGAP1* [33], and *TBC1D1* genes [34] have been identified. Although these results did not include our candidate SNPs, we assumed that this discrepancy was related to our focus on cases of CAKUT accompanied by cryptorchidism. Genome-wide analyses have made it possible to investigate multiple gene loci simultaneously. Chatterjee et al. investigated mutations in several genes involved in the GDNF-RET signaling pathway and subsequently detected double non-synonymous variants of RET (G691S/R982C) in a patient with complex CAKUT and cryptorchidism [13]. They also reported that the CAKUT and cryptorchidism phenotypes could be explained by the occurrence of a combination of rare, novel, and common deleterious variants affecting the RET pathway. However, the complete pathological mechanism can only be explained by genome-wide analyses because monogenic causes are detected in only 5–12% of patients [5, 13]. Exons account for only 2% of the whole genome, and the roles of noncoding regions or epigenetics in CAKUT remain unclear. Indeed, we have previously demonstrated that copy number variations in the region upstream of *SOX3*, suspected to act as a promoter or enhancer, are associated with testicular differentiation [14].

The present study had several limitations. First, the sample size used in the study was too small to investigate a specific phenotype. Moreover, we did not determine whether genetic variations in these eight genes resulted in a dysfunction at the protein level. We investigated the effects of the identified amino acid substitutions on protein function using the SIFT platform (http://sift.jcvi.org/). All 10 SNPs detected in the present study were predicted to be tolerant in the in silico analysis (data not shown). Besides, MAF of SNPs in *SMAD4* and *FREM2* (rs2496423) is lower than 1%, suggesting that these rare variants is possibly pathogenic for CAKUT accompanied with by cryptorchidism. Further investigations are needed to determine whether other factors associated with the BMP/SMAD signaling pathway are involved in the onset of CAKUT with cryptorchidism.

## Conclusions

We detected 10 non-synonymous SNPs in eight genes in patients with CAKUT accompanied by cryptorchidism. These SNPs were candidate polymorphisms associated with the development of the kidney, urinary tract, and testis. BMP8B is known to activate the nuclear translocation of SMAD4, which regulates the expression of genes associated with the differentiation of primordial germ cells or testicular development. Additionally, BMP4, a member of the BMP family, regulates the interaction between the metanephric mesenchyme and ureteric bud by suppressing GDNF. Taken together, these findings suggested that the developmental mechanisms of the kidneys and urinary tract were intimately linked with that of the male reproductive organs through genetic alterations.

## Abbreviations

CAKUT: Congenital anomalies of kidney and urinary tract
SNP: Single nucleotide polymorphism
WES: Whole exome sequencing
